# Cocaine use during pregnancy assessed by hair analysis in a Canary Islands cohort

**DOI:** 10.1186/1471-2393-12-2

**Published:** 2012-01-09

**Authors:** Xavier Joya, Mario Gomez-Culebras, Alicia Callejón, Bibiana Friguls, Carme Puig, Sandra Ortigosa, Luca Morini, Oscar Garcia-Algar, Oriol Vall

**Affiliations:** 1 Unitat de Recerca Infància i Entorn (URIE), Institut de Recerca Parc de Salut Mar (IMIM-Parc de Salut Mar), Barcelona, Spain; 2 Red de Salud Materno-Infantil y del Desarrollo (SAMID; 3 Programa RETIC, Instituto Carlos III (ISCIII), Madrid, Spain; 4 Departamento de Cirugía Pediátrica, Universidad de Tenerife, Spain; 5 Servei de Pediatria, Parc de Salut Mar, Barcelona, Spain; 6 Departament de Pediatria, Ginecologia i Obstetrícia i Medicina Preventiva, Universitat Autònoma de Barcelona (UAB), Barcelona, Spain; 7 Department of Legal Medicine and Public Health, University of Pavia, Italy

## Abstract

**Background:**

Drug use during pregnancy is difficult to ascertain, and maternal reports are likely to be inaccurate. The aim of this study was to estimate the prevalence of illicit drug use among pregnant women by using maternal hair analysis.

**Methods:**

A toxicological analysis of hair was used to detect chronic recreational drug use during pregnancy. In 2007, 347 mother-infant dyads were included from the Hospital La Candelaria, Santa Cruz de Tenerife, Canary Islands (Spain). Data on socioeconomic characteristics and on substance misuse during pregnancy were collected using a structured questionnaire. Drugs of abuse: opiates, cocaine, cannabinoids and amphetamines were detected in maternal hair by immunoassay followed by gas chromatography-mass spectrometry for confirmation and quantitation.

**Results:**

Hair analysis revealed 2.6% positivity for cocaine and its metabolites. Use of cocaine during pregnancy was associated with unusual behaviour with potentially harmful effects on the baby.

**Conclusions:**

The results of the study demonstrate significant cocaine use by pregnant women in Canary Islands. The data should be used for the purpose of preventive health and policy strategies aimed to detect and possibly to avoid in the future prenatal exposure to drugs of abuse.

## Background

As documented by the European Monitoring Centre on Drugs and Drug Addiction (EMCDDA), drug use in Spain shows a growing trend. The prevalence for cocaine use appear to be at 3% [1], exceeding the rates reported from USA [1,2]. When cocaine is used during pregnancy, it can affect the cardiac and vascular systems of both, the pregnant woman and the foetus. The effects of cocaine on the foetal-placental unit can lead to placental detachment or diminished blood flow with subsequent hypoxia [3-5]. This could explain the frequency of spontaneous abortions and foetal deaths observed in cocaine abusing mothers [6].

The information in the literature regarding the prevalence of recreational drug use, specifically in pregnant women is limited. Studies carried out in USA estimate the rates of infants exposed prenatally to illicit drugs to be between 6% and 40% of all live births [7-11]. In Spain, the single large study focused on prenatal exposure to drugs of abuse was conducted in Barcelona (alcohol, tobacco and prescription drugs were not included); using meconium testing an overall positivity of 10.9% for any drug of abuse was shown [12].

It is well known that maternal self-reports on drug use history proved to be unreliable therefore an objective biological marker which can yield a cumulative reflection of long-term exposure to illicit drugs is needed. Since drugs and their metabolites are permanently deposited in the protein matrix of the hair, they can be detected in hair several months after use, which provides an advantage over other biological markers, such as urine or blood which are limited to present only a "snapshot" of acute exposure to drugs in the previous 24-48 hours [13-15]. Moreover, maternal hair analysis allows for drug use studies in the first trimester of pregnancy and has proven to be more sensitive and stable compared to other matrices with large exposure windows, such as meconium or neonatal hair [16-19]. Acquiring maternal hair is easy and availability is a non-issue.

Prenatal exposure to drugs of abuse is, of course, dependent on maternal use during pregnancy. The exposure data is different in every area, depending on socioeconomic and geographical factors. Prevalence of prenatal drug exposure in the Canary Islands is unknown. These islands are located in the Atlantic Ocean, about 100 km from the nearest point of the North African coast (south west of Morocco). The economy of Canary Islands is based on a few sectors: tourism and, to a much lesser extent, farming and fishing. Tenerife, the largest and most populated of the seven Canary Islands contains 43% of the total population of the Canary Islands and it is well known for being a holiday resort with a thriving night life, associated with clubs, music and recreational drugs use.

The aim of this study was to estimate the prevalence of illicit drug use among pregnant women in Tenerife by using maternal hair analysis.

## Methods

### Subjects and samples

The study was carried out in the paediatric department of the Hospital La Candelaria of Santa Cruz de Tenerife, Spain, the main hospital of the island. Between June 2006 and June 2007, 347 mother-infant dyads agreed to participate in the study, without any refusal. Fifteen dyads were not included due to different reasons: language issues, death of newborn, critical illness of the mother. (N = 347). The study was approved by the Institutional Ethical Committee, conducted in accordance with the Declaration of Helsinki and signed informed consent was obtained from participant parents. Parental socio-demographic characteristics, life habits and drug habits during pregnancy were recorded in previously validated questionnaires administered to all participants the day after the delivery (Additional file 1) [10-12,15,17,20]. Obstetric history of the mother was collected and neonatal anthropometric characteristics using customised growth charts for birth weight and height and clinical examination at birth were also recorded.

On the day of delivery, a lock of scalp hair approximately 0.5 cm thick was cut from the posterior vertex of the mothers as closely as possible to the skin. Since hair grows at an approximate rate of 1 cm/month, in order to document exposure in the last trimester of pregnancy, the proximal segment (3 cm long) of the hair was considered for the analysis of drugs of abuse. Hair was collected in accordance with the SoHT (Society of Hair Testing) recommendations and it was stored at room temperature in a paper bag until analysis [21].

### Determination of drugs of abuse in hair samples

Drugs of abuse including all the principal psychotropic drugs and metabolites (6-monoacetylmorphine (6-MAM), morphine and codeine for opiates; cocaine, benzoylecgonine (BEG) and cocaethylene for cocaine; Δ9-tetrahydrocannabinol (THC) and 11-nor-Δ9-tetrahydrocannabinol-9-carboxylic acid (THC-COOH) for cannabinoids; and amphetamine, methamphetamine, and 3,4 methylenedioxyamphetamine (MDMA) for amphetamines were quantified.

Immunochemical screening for drugs of abuse in hair was done with Siemens EMIT^® ^II Plus assay for opiates, amphetamines and ecstasy and Microgenics CEDIA^® ^assay for cocaine. All the samples with positive results in the screening test (the cut-off values for opiates, amphetamines, ecstasy, cocaine, and THC (directly, not included in the previous screening) were 0.20 ng/mg, 0.50 ng/mg, 0.50 ng/mg, 0.20 ng/mg, and 0.50 ng/mg respectively) for any drug and/or metabolite were analyzed by gas chromatography-mass spectrometry (GC/MS) for confirmation and quantitation [9,15,21-23].

GC/MS analyses were carried out on an Agilent 6890 gas chromatograph equipped with 5973 mass-selective detector and a 7673 automatic injector (Agilent Technologies, Palo Alto, CA, USA). Limits of quantification in hair were: 0.2 ng/mg for 6-monoacetylmorphine, morphine and codeine, 0.5 ng/mg for cocaine, BEG and cocaethylene; 0.1 ng/mg for Δ9-tetrahydrocannabinol, 0.2 pg/mg 11-nor-Δ9-tetrahydrocannabinol-9-carboxylic acid and 0.2 ng/mg for amphetamine, methamphetamine, and MDMA.

### Statistical analysis

The continuous variables that followed a normal distribution were presented as mean and standard deviation. The categorical variables were presented as percentages and were compared between variables with the Chi-square. P value of less than 0.05 (two-tail) was considered to be statistically significant.

An initial descriptive statistical analysis of socio-demographic characteristics of mothers and anthropometric data of the newborns was completed for the 347 mother-infant dyads participating in the study. The group of positive mother-infant dyads was compared with the group of negative mother-infant dyads for any drug. Statistical analysis was performed considering the drug use (yes or no) as the dependent variable. For the bivariate analysis of the data we used the Student's t test for independent groups to compare between means of a continuous variable and the Chi-square test to compare between categorical variables. Statistical significance was set at *p *< 0.05. Database management and statistical analysis were performed with SPSS v 14.0 (SPSS, Chicago, IL, USA).

## Results

### Socio-demographic characteristics and obstetric history

The mean age of the 347 mothers was 30 years. Seventeen percent were migrants, 85% from South-America. With respect to the level of schooling, 29% had a university degree and 22% had only primary schooling. Regarding obstetric history, 49% were at their first pregnancy, and 22% had previous spontaneous abortions.

Parental socioeconomic and demographic characteristics in relation to results obtained by hair analysis are reported in Table 1 Parental ethnicity and maternal or paternal socioeconomic status were not associated with cocaine use during pregnancy, but cocaine use appeared to be more prevalent in Spanish mothers with unskilled jobs. On the other hand, the exposure to cocaine during pregnancy was associated with a significantly higher percentage of active tobacco smoking fathers and the presence of other smokers at home. There were no false negatives in our study population comparing biomarkers to questionnaires (admission of substance misuse). Finally, cocaine use during pregnancy was associated with antidepressants use.

**Table 1:** Parental Socio-demographics and exposure to drugs of abuse during pregnancy

	Mother's hair positive for cocaine and/or BEG (N = 9)	Mother's hair negative for any drug of abuse (N = 365)
**Parental Nationality**		

**Mother's nationality**		

Spanish	9 (100.0%)	298 (81.7%)

Non-Spanish	0 0%)	67 (18.2%)

**Father's nationality**		

Spanish	9 (100.0%)	298 (81.7%)

Non-Spanish	0 (0%)	67 (18.2%)

**Maternal age **(years), mean (S.D.)	28.6 (6.0)	30.3 (5.3)

**Mother's Educational Level**		

Unfinished elementary school	4 (44.4%)	85 (23.2%)

**Employed mother (yes/no)**		

No	2 (22.2%)	110 (30.1%)

**Mother's socioeconomic status**		

Managerial, professional & skilled (non-manual)	3 (33.3%)	101 (28.0%)

Skilled (manual) and partly skilled	5 (55.6%)	224 (61.3%)

Unskilled	1 (11.1%)	40 (10.9%)

**Single Mother**		

Yes	0 (0%)	12 (3.3%)

**Father's Educational Level**		

Unfinished elementary school	4 (44.4%)	120 (33.2%)

**Employed father (yes/no)**		

No	0 (0%)	25 (7.0%)

**Father's socioeconomic status**		

Managerial, professional & skilled (non-manual)	1 (11.1%)	101 (28.0%)

Skilled (manual) and partly skilled	7 (77.8%)	244 (66.9%)

Unskilled	1(11.1%)	20 (5.1%)

**Habitat**		

Rural (< 10.000 inhab.)	1 (11.1%)	56 (15.3%)

Semi-rural (10 - 100.000 inhab.)	5 (55.6%)	127 (34.7%)

Urban (> 100.000 inhab.)	3 (33.3%)	182 (50.0%)

**Maternal drug use at the visit**		

Past substance misuse, yes	5 (55.6%)**	47 (12.8%)

Cocaine use, yes	4 (44.4%)**	15 (4.2%)

**Tobacco smoking**		

Mother	7 (77.8%)*	60 (16.3%)

Number of daily cigarettes, mean (S.D.)	6.5 (2.1)	7.2 (6.0)

Did you smoke before pregnancy?, yes	8 (88.8%)*	100 (27.3%)

Father	7 (77.8%)*	138 (37.9%)

Have been exposed to tobacco smoke during pregnancy?	4 (44.4%)	101 (28.0%)

Other smokers in the presence of the pregnant woman?	2 (22.2%)*	23 (6.3%)

**Use of Antidepressants**, yes	(11.1%)*	8 (2.2%)

**Alcohol use**, yes	1 (0%)	15 (4.2%)

** P < 0.005 respect control group

* P < 0.05 respect control group

### Concentrations levels of drugs of abuse in hair from pregnant women

Of the 347 maternal hair samples minimum 3 cm long, 9 (2.6%) were positive for cocaine and/or BEG, its principal metabolite. All the samples were negative for the other drugs of abuse including cannabis, amphetamines, methamphetamines, opiates, and MDMA.

Cocaine concentration in hair ranged between 0.25 and 2.06 ng/mg with a median value of 1.1 ng/mg and BEG concentration ranged between 0.31 and 3.18 ng/mg with a median value of 1.55 ng/mg. Benzoylecgonine/cocaine ratios ranged between 0.05 and 1.61 with a median value of 0.84.

### Obstetric history and newborn anthropometric characteristics at birth

The obstetric history and newborn anthropometric characteristics are presented in Table 2. Maternal age was not associated with substance misuse. Drug using mothers had a higher number of previous abortions. There were no significant differences with respect to newborn's sex between the positive and negative groups, but there was a trend of more female newborns in both groups. Newborns from cocaine users showed lower birth weight, crown-heel length and cranial perimeter than newborns from non-using mothers, but these differences did not reach significance. There was no withdrawal syndrome or malformation in any of the drug exposed newborns.

**Table 2:** Anthropometric Characteristics of the newborns according to the results obtained

	Mother's hair positive for cocaine and/or BEG (N = 9)	Mother's hair negative for any drug of abuse (N = 365)
**Previous pregnancies**		

No	4 (44.4%)	177 (48.5%)

1	2 (22.2%)	120 (32.8%)

> 2	3 (33.3%)	58 (15.9%)

**Previous premature infants**		

Yes	0 (0%)	3 (0.9%)

**Previous abortions**		

Yes	4 (44.4%)	85 (23.2%)

**Children characteristics at birth**		

Gender, female	2 (22.2%)	193 (52.8%)

Gestational age (weeks), mean (S.D.)	38.9 (1.1)	38.9 (1.5)

Prematurity	0 (0%)	25 (6.9%)

Weight at birth (g), mean (S.D.)	3199 (523)	3288 (499)

Length at birth (cm), mean (S.D.)	49.8 (2.6)	50.8 (2.7)

Cranial perimeter (cm), mean (S.D.)	33.4 (1.6)	34,2 (1,5)

**Outcomes at birth**		

Yes	3 (33.3%)	125 (34.1%)

Loss of foetal well-being	0 (0%)	4 (1.2%)

Risk of perinatal infection	3 (33.%)4	72 (19.8%)

Hypoglycaemia	0 (0%)	23 (6.3%)

Developmental dysplasia of the hip	0 (0%)	5 (1.5%)

Other outcomes †	0 (0%)	6 (1.8%)

** P < 0.005 respect control group

* P < 0.05 respect control group

† including respiratory, cardiac and dermatological diseases

## Discussion

### Prevalence of substance misuse among pregnant women

It is very difficult, based on the literature, to compare the prevalence of substance misuse by pregnant women in different populations for many reasons: studies may relate to different time periods, biological matrices have different windows of detection and there are specific patterns of substance misuse in different countries [24].

The European trend of increased cocaine use in the general population, including pregnant women, could also be observed in Tenerife. The results obtained in this study are very similar to the results obtained in Barcelona [12,20] and Glasgow (UK) [25] using meconium analysis.

Prevalence of drug exposure in our study is compared to the results reported in other population studies [12,20,25-28] and the results of the Spanish National Survey on Drug Abuse [1] in Table 3.

**Table 3:** Comparison of the prevalence of drug exposure in our study population with the one reported in the questionnaire-based PNSD and the results reported by other population

	**Sherwood et al**. [25]	**Pichini et al**. [12,22]	**Williamson et al**. [23]	**Mitsuhiro et al**. [24]	**PNSD **[1]	**Friguls et al **[26]	**Joya et al**.
**Year**	**1999**	**2005**	**2006**	**2007**	**2009**	**2010**	**2011**

**Place**	London (UK)	Barcelona (Spain)	Glasgow (UK)	Sao Pablo (Brazil)	Spain	Ibiza (Spain)	**Tenerife (Spain)**

**Matrix**	Urine	Meconium	Meconium	Hair	Questionnaire	Maternal hair	**Maternal hair**

**Trimester**	1st	2nd - 3rd	2nd - 3rd	3rd	No pregnant	3rd	**3rd**

**Population**	general	Low-income	Low-income	Low-income	general	general	**general**

**N**	807	830	400	1000	23715	107	**347**

**Cannabis (%)**	14.5	5.3	13.2	4.0	13.2	10.3	**0**

**Cocaine (%)**	0.37	2.6	2.7	1.7	3.0	6.4	**2.6**

**MDMA (%)**		0.1			1.4	0.9	**0**

**Opiates (%)**	1.36	4.7			0.1	0	**0**

**Any drug (%)**	16.2	10.1		6.0		15.9	**2.6**

Hair testing as an analytical tool allowed for a more accurate identification of newborns exposed in utero to drugs of abuse compared to the identification based only on maternal questionnaire. Our findings are in agreement with the well-known under-reporting of tobacco smoke and drug use by pregnant women, as documented in several studies [11,26]. Overall, these data confirm the steady cocaine use showed by EMCDDA using questionnaires [1].

Surprisingly, we did not find any samples positive for cannabis, in spite of the fact that it is the most abused drug in European countries. According to data from the Canarias Government, daily use of cannabis by adult women in Tenerife is 1.0% [27]. It is worth mentioning that, in theory, a single exposure to drugs could lead to a positive result in hair analysis, but this occurrence has been demonstrated in only 1 experiment in adults after the administration of intravenous/intranasal cocaine, but certainly not with cannabis use [28].

Finally, no opiates were detected in the Tenerife population in contrast to the 4.7% opiates found in the Barcelona study population. This might be due to the fact that the population studied in Barcelona was from a low socio-economic status using frequently a mixture of smoked heroin and cocaine [29].

No positive cases of maternal alcohol use were recorded by questionnaire in the exposed group of babies. This is in agreement with the results of other studies based on questionnaires, however, based on biomarkers, it has been shown that prenatal exposure to alcohol could reach 43% [30].

### Characteristics of the drug users and of the newborns exposed in utero to drugs

Even though substance misuse has been associated with low income and lack of education [31], we found no associations with the level of schooling or profession. Our study showed that drug using mothers present with behavioural patterns with potentially harmful effects on the child's health (e.g. tobacco smoking, benzodiazepines and/or antidepressants use) to a significantly higher extent. Data from our study highlighted a higher rate of pre-pregnancy drug use (declared by questionnaire) among tobacco smoking women compared to non-smoking women. This tendency has been already reported in pregnant women [12] and in the general population [32]. We also found an association between maternal drug use during pregnancy and maternal and paternal tobacco smoking. Also, in our study, the correlation between cocaine use and concurrent antidepressants use was significant. This association was reported on previously by Joya et al [19] The finding requires a close follow-up of these newborns [33] because of the high risk of withdrawal syndrome and adverse effects due to prenatal exposure not only to cocaine but antidepressants as well.

According to some studies, prenatal exposure to cocaine seems to have a negative effect on the child's neurological development and intellectual and emotional behaviour, due to the effect of cocaine on the monoaminergic system [34,35]. In our study, on the basis of self-reporting, the newborns exposed to cocaine did not show (significant) differences on somatometry characteristics. However, it is well documented that ongoing maternal and environmental risk factors (e.g. drug abuse, violence, poor child care, and maternal depressive symptoms) have been associated with worse developmental outcomes in prenatally exposed children [36,37]. The higher number of previous abortions may be due to problem behaviour and lack of family planning in the case of these women [38].

### Hair is a useful matrix to evaluate chronic substance misuse during pregnancy

Even though 2.6% of the mothers had positive results for cocaine use during the third trimester of pregnancy, less than half of the mothers admitted drug use during pregnancy by questionnaire. This is not surprising, since the tendency to under-report recreational drug use by pregnant women was highlighted previously [12,15,18]. Hair testing for drugs provides a wide window of detection and sample collection is not invasive [39]. GC/MS proved to be a highly sensitive and specific technique for the detection of low concentrations of drugs [20,40]. Some authors [14,15] concluded that, compared to meconium analysis, hair analysis has higher sensitivity for detecting prenatal use of cocaine and opiates. The method of maternal hair analysis also allows for a more accurate estimation of the timing of drug use compared to neonatal hair testing, as neonatal hair has an irregular prenatal growth rate [24]. In spite of the fact that hair testing is very useful in prevalence studies on prenatal exposure to drugs of abuse, there are very few published reports to date on this topic.

### Interventions for a pregnancy free of drugs of abuse and for follow-up of prenatally exposed newborns

The results of our study justify for future screening for drug use during pregnancy, that could provide the necessary proof to start treatment for substance abuse which is significantly more effective during pregnancy than in other periods in a woman's life. Early recognition, early intervention, timely enrolment into treatment, and a sustained, long-term treatment regimen could minimize the foetal impact of perinatal maternal illicit drug use and may improve a woman's prognosis for successful, ongoing recovery from addiction. The development of a screening and intervention protocol will undoubtedly help medical care providers to make objective decisions regarding their screening/testing/intervention practices for women with substance abuse issues during pregnancy and for their offspring [12,20]. We must emphasize that these results are very important for public health and we suggest some actions in order to be informed about the prevalence of prenatal exposure to drugs of abuse and to implement specific programs such as counselling with brief intervention during pregnancy to avoid maternal substance misuse and screening interventions during pregnancy and at birth.

### Limitations

The samples came from one single hospital, however, the Hospital La Candelaria in Tenerife has the largest number of births on the island, and by extension the entire archipelago. There are no results for prenatal exposure to alcohol as the main legal drug of abuse, but the samples are in the process of being analyzed and the results will be presented.

Finally, hair analysis results could have been compared to meconium analysis results as another standard tool to detect prenatal chronic exposure to drugs of abuse. Meconium is a direct neonatal biological matrix, but is informative only about the second (?) and third trimester of pregnancy. Conversely, a sufficiently long maternal hair will allow for information on the entire prenatal period; in our study we used only the proximal 3 cm. segment of hair.

## Conclusions

These data underline the usefulness of hair analysis for the diagnosis of chronic drug use during pregnancy and demonstrate that there is significant hidden, undeclared use of cocaine by pregnant women in the Canary Islands that may cause multiple complications for both the baby and the mother. The data can be used for the purpose of preventive health and policy strategies aimed to avoid and to detect prenatal exposure to drugs of abuse. Therefore, it is essential to implement specific counselling and to introduce screening methods during pregnancy. Finally, uniform guidelines should be provided for health and social service professionals.

## Competing interests

The authors declare that they have no competing interests.

## Authors' contributions

XJ, was the main contributor in writing the manuscript. MGC, analyzed the mother-infant data, reviewed the literature and the final manuscript, and contributed in writing the manuscript. AC, was the main contributor to the recruitment, and contributed in writing the manuscript. BF, analyzed the mother-infant data, and was a contributor in writing the manuscript. CP, analyzed the mother-infant data, and was the statistical expert. SO, analyzed the mother-infant data, reviewed the literature and the final manuscript. LM, was the main laboratory technician in biomarkers analyses, and contributed in writing the manuscript. OGA, analyzed the mother-infant data, reviewed the literature, and was a major contributor in writing the manuscript. OV, was the paediatrician responsible for coordination of data, and contributed in writing the manuscript. All the authors read and approved the final manuscript.

## Pre-publication history

The pre-publication history for this paper can be accessed here:

http://www.biomedcentral.com/1471-2393/12/2/prepub

## Supplementary Material

Additional file 1 **Questionnaire**. Questionnaire administered to all participants the day after the delivery. Click here for file
