# Supplementary material for: Cocaine use during pregnancy assessed by hair analysis in a Canary Islands cohort
Source: BMC Pregnancy Childbirth. 2012 Jan 9;12:2. doi: 10.1186/1471-2393-12-2 (PMC3277455; doi:10.1186/1471-2393-12-2)
Supplement: Additional file 1 — Questionnaire. Questionnaire administered to all participants the day after the delivery. [file 1471-2393-12-2-S1.DOC]

#### QUESTIONNAIRE

NUM:

COCAINE USE DURING PREGNANCY ASSESSED BY HAIR ANALYSIS IN A CANARY ISLANDS COHORT

DATE:

1. DEMOGRAPHIC DATA

Mother

Age

Ethnic origin

Nationality (Spanish, non-Spanish)

Country

Employed mother

Educational level (unfinished elementary school)

Mother’s socioeconomic status (managerial, professional & skilled (non-manual), skilled (manual) and partly skilled, unskilled)

Single mother

Habitat (rural (< 10.000 inhab.), semi-rural (10 – 100.000 inhab.), urban (> 100.000 inhab.))

Father

Age

Ethnic origin

Nationality

Country

Employed father

Educational level (unfinished elementary school)

Father’s socioeconomic status (managerial, professional & skilled (non-manual), skilled (manual) and partly skilled, unskilled)

Habitat (rural (< 10.000 inhab.), semi-rural (10 – 100.000 inhab.), urban (> 100.000 inhab.))

2. OBSTETRIC DATA

Parity

Gestational age

Birth date

Previous pregnancies

Previous premature infants

Previous abortions

3. NEONATAL DATA

Gender

Gestational age (weeks)

Prematurity

Weight at birth (g)

Length at birth (cm)

Craneal perimeter (cm)

Outcomes at birth

Loss of fetal well-being

Risk of perinatal infection

Hypoglycemia

Developmental dysplasia of the hip

Other outcomes

4. MATERNAL DRUGS USE AT THE VISIT

Past drugs of abuse consumption

Cocaine use

Cannabis use

Heroin use

Methadone use

Amphetamines use

Other drugs of abuse

How many times every day/week?

The same all the months?

Use of antidepressants

Use of others drugs of prescription

Alcohol use

How many drinks per day/per week?

The same drinks all the months?

5. TOBACCO SMOKING

Mother

Did you smoke before pregnancy?

During pregnancy?

The same number of cigarrettes all the months?

Number of daily cigarettes

Have been exposed to tobacco smoke during pregnancy?

Father

Did he smoke during pregnancy?

Number of daily cigarettes

Other smokers in the presence of the pregnant woman?
